# Immuno-Therapy with Anti-CTLA4 Antibodies in Tolerized and Non-Tolerized Mouse Tumor Models

**DOI:** 10.1371/journal.pone.0022303

**Published:** 2011-07-14

**Authors:** Jonas Persson, Ines Beyer, Roma Yumul, ZongYi Li, Hans-Peter Kiem, Steve Roffler, André Lieber

**Affiliations:** 1 Department of Medicine, University of Washington, Seattle, Washington, United States of America; 2 Fred Hutchinson Cancer Research Center, Seattle, Washington, United States of America; 3 Institute of Biomedical Sciences, Academia Sinica, Taipei, Taiwan; 4 Department of Pathology University of Washington, Washington, United States of America; French National Centre for Scientific Research, France

## Abstract

Monoclonal antibodies specific for cytotoxic T lymphocyte-associated antigen 4 (anti-CTLA4) are a novel form of cancer immunotherapy. While preclinical studies in mouse tumor models have shown anti-tumor efficacy of anti-CTLA4 injection or expression, anti-CTLA4 treatment in patients with advanced cancers had disappointing therapeutic benefit. These discrepancies have to be addressed in more adequate pre-clinical models. We employed two tumor models. The first model is based on C57Bl/6 mice and syngeneic TC-1 tumors expressing HPV16 E6/E7. In this model, the HPV antigens are neo-antigens, against which no central tolerance exists. The second model involves mice transgenic for the proto-oncogen *neu* and syngeneic mouse mammary carcinoma (MMC) cells. In this model tolerance to *Neu* involves both central and peripheral mechanisms. Anti-CTLA4 delivery as a protein or expression from gene-modified tumor cells were therapeutically efficacious in the non-tolerized TC-1 tumor model, but had no effect in the MMC-model. We also used the two tumor models to test an immuno-gene therapy approach for anti-CTLA4. Recently, we used an approach based on hematopoietic stem cells (HSC) to deliver the relaxin gene to tumors and showed that this approach facilitates pre-existing anti-tumor T-cells to control tumor growth in the MMC tumor model. However, unexpectedly, when used for anti-CTLA4 gene delivery in this study, the HSC-based approach was therapeutically detrimental in both the TC-1 and MMC models. Anti-CTLA4 expression in these models resulted in an increase in the number of intratumoral CD1d+ NKT cells and in the expression of TGF-β1. At the same time, levels of pro-inflammatory cytokines and chemokines, which potentially can support anti-tumor T-cell responses, were lower in tumors of mice that received anti-CTLA4-HSC therapy. The differences in outcomes between the tolerized and non-tolerized models also provide a potential explanation for the low efficacy of CTLA4 blockage approaches in cancer immunotherapy trials.

## Introduction

Activation of T-cells requires recognition of antigens presented in complex with CD80 and CD86. These costimulatory molecules interact with CD28, which is constitutively expressed on T cells and triggers T-cell activation. Once activated, T-cells transiently up-regulate cytotoxic T lymphocyte–associated antigen 4 (CTLA4) on their cell surface. CTLA4 shares structural features with the costimulatory receptor CD28 and reciprocally targets the same costimulatory molecules (CD80/86) on the antigen-presenting cell, but with higher affinity. This results in inhibition of T-cell proliferation and IL-2 production. Blocking CTLA4 with anti-CTLA4 antibodies enhances effector T-cell responses and can induce T-cell mediated rejection of certain tumors in mouse models [1], [2], [3], [4]. Monoclonal antibodies specific for cytotoxic T lymphocyte-associated antigen 4 (CTLA4) are a form of experimental immunotherapy for treatment of patients with advanced cancers, including melanoma, prostate cancer, renal cell carcinoma, non-Hodgkin's lymphoma, colorectal carcinoma, non-small lung breast cancer, and pancreatic cancer [5]. Two fully humanized monoclonal antibodies, ipilimumab (MDX-010, Medarex) and tremelimumab (CP-675,206, Pfizer), have been investigated in cancer [6], [7]. A Phase III trial of tremelimumab has been halted after it failed to demonstrate superior therapeutic activity over standard chemotherapy in advanced melanoma patients. The discrepancy in pre-clinical and clinical studies with anti-CLTA4 antibodies requires more mechanistic studies in adequate pre-clinical models. A potential mechanism by which anti-CTLA4 may provide an antitumor response is through depletion of regulatory T-cells (Tregs), as Tregs have constitutive expression of CTLA4 and are known to have suppressive activity. Alternatively, CTLA4 blockade may activate effector T-cells allowing them to be more resistant to Treg suppression. Recent studies indicate that anti-CTLA4 induce immune responses mainly by direct activation of effector T-cells rather than by affecting Tregs [8], [9].

In this study, we used two tumor models that assess anti-CTLA4 antibody therapy. The first is a murine cervical cancer model based on human papillomavirus (HPV)-16 E6/E7–expressing TC-1 tumors. In this model, the HPV antigens represent neo-antigens against which no central tolerance mechanisms exit in mice. Most studies on the mechanisms of immune-activation by CTLA4-blocking antibodies have been performed in such “non-tolerized” models [10], [11], [12], [13]. In humans, however, most tumor-associated antigens (TAAs) are non-mutated self-antigens, which are overexpressed or re-expressed on cancer cells. Several mechanisms of central and peripheral tolerance therefore exist against self-TAAs that blunt T-cell responses. Tolerance against TAA has to be considered in tumor models that are used to delineate the anti-tumor mechanisms of anti-CTLA4 antibodies. This is accomplished in our second animal model, based on *neu*-transgenic (*neu*-tg) mice. These mice overexpress the rat protooncogene *Neu* and develop spontaneous mammary tumors between 4 and 8 months of age [14], [15]. Mouse mammary carcinoma cells (MMC) are a transplantable carcinoma line derived from a spontaneous mammary tumor from *neu*-tg mice. The *neu*-tg/MMC model has significant biologic and pathologic similarity to human *neu*-associated estrogen receptor-negative breast cancer. MMC tumors are resistant to doxorubicin, hormone therapy, and *Neu*-specific mAbs. The tumor antigen repertoire in MMC-tumor bearing mice appears to be predictive for human breast cancer antigens. Importantly, *neu-*tg mice mimic central/peripheral tolerance to an endogenous tumor antigen that is seen in cancer patients. In this context, *Neu*-targeted vaccines, which raise strong CD8-T cell responses to a dominant peptide (RNEU420-429) in (non-tolerized) WT FVB/N mice and protect them from a *neu*-expressing tumor challenge, fail to do so in *neu*-transgenic mice. The latter suggests significant differences between tolerized and non-tolerized tumor models, which have to be considered in testing the effect of new immunotherapy agents.

For delivery of anti-CTLA4 immunotherapy, we used three different approaches; *i)* systemic application of a monoclonal antibody against murine CTLA4 (4F10), *ii)* intratumoral expression of a secreted form of this antibody from genetically modified tumor cells, *iii)* expression of the anti-CTLA4 antibody after gene delivery using a stem cell based approach.

The central findings from our studies are *i)* anti-CTLA4 therapy is inefficient in the tolerized MMC model and *ii)* in both tumor models, anti-CTLA4 expression mediated by the HSC delivery approach not only failed to exert anti-tumor effects, but increased the rate of tumor growth. Our data suggests that the latter involves an increase in intratumoral CD1d+ NKT cells, production of IFNβ1, as well as suppression of cytokines and chemokines that are involved in mediating anti-tumor immune responses. Our findings shed light on the complexity of immune regulation, specifically in the context of anti-CTLA4 therapy.

## Materials and Methods

### Anti-CTLA4 antibodies

Monoclonal antibodies against mouse CTLA4 were purified from the supernatant of UC10-F10-11 hybridoma cells (ATCC) as described previously [16].

### Lentivirus vectors

The anti-CTLA4 scFv was cloned from total RNA isolated from UC10-F10-11 hybridoma cells (ATCC). Leucine residues at positions 43 and 89 in the 4F10 variable region light chain sequence were mutated to methionine and glutamine, respectively, to increase scFv expression [17]. The anti-CTLA4 scFv was inserted into pHook (Invitrogen, Carlsbad, CA) immediately after the Vκ leader sequence and HA epitope tag and before the myc epitope and platelet-derived growth factor receptor transmembrane domain. A stop codon was introduced immediately after the myc epitope to allow secretion of the antibody. The cDNA fragment coding the hinge-CH_2_-CH_3_ of human IgG_1_ was inserted between the scFv and myc epitope. The entire anti-CTLA4 cassette was then transferred into pLVPT-rTRKRAB [18] to generate pLVaCTLA4. To create the construct for the insulated vector (I-LV-aCTLA4), the aCTLA-4, IRES, tetR-KRAB and WPRE from pLVaCTLA4 was transferred into pLenti-cHS-PGK [19] containing a 0.4 kb fragment of the chicken HS4 insulator within the 3′ LTR. VSV-G pseudotyped viruses were generated as described earlier [20]. Genome titers were measured by qPCR and ranged from 1-5×10^7^ genomes/ml.

### Cells

Mouse mammary carcinoma (MMC) cells were established from a spontaneous tumor in a *neu*-tg mouse [21]. TC-1 cells were from the American Type Culture Collection (ATCC) Culture conditions for MMC and TC-1 cells were RPMI-1640 medium containing 10% fetal bovine serum (FBS), 2 mM L-glutamine (Gln), 100 U/ml penicillin (P), and 100 µg/ml streptomycin (S).

To obtain mouse HSCs, donor mice were injected with 5-FU (150 mg/kg) i.v. two days before bone marrow isolation. Bone marrow cells were cultured for three days in IMDM, 18%FBS, 5% mIL-3 (BD Biosciences, San Jose, CA), 100 U/ml mIL-6, 50 U/ml SCF (PeproTech, Rocky Hill, NJ), P/S, and Gln. Non-adherent cells were collected and incubated with lentivirus vectors at an MOI of 2 genomes/cell on retronectin-coated plates for two days.

### Animal studies

All experimental procedures involving animals were conducted in accordance with the institutional guideline set forth by the University of Washington. *Neu*-transgenic (*neu*-tg) mice [FVB/N-Tg(MMTV*neu*)202 Mul] were obtained from the Jackson Laboratory (Bar Harbor, ME). These mice harbor non-mutated, non-activated rat *neu* under control of the mouse mammary tumor virus (MMTV) promoter. The *neu* transgene is expressed at low levels in normal mammary epithelium, salivary gland, and lung. Until the age of 8 months ∼35% of female *neu*-tg mice spontaneously develop mammary carcinomas that display high *Neu*-expression levels. CTLs specific for the immunodominant H-2 Dq/RNEU_420–429_ epitope can be detected in *neu*-tg mice using the corresponding tetramer [22]. For HSC transplantation, a total of 1×10^6^ of whole bone marrow cells or lineage cell depleted bone marrow cells from 5-FU treated mice were transplanted into lethally irradiated (1050 cGy) female *neu*-tg mice. Six weeks after bone marrow transplantation, the mice received 5×10^5 ^MMC or 5×10^4^ TC-1 cells via subcutaneous injection. Tumors were measured every other day and tumor volume was calculated as the product of length x width x width. For survival studies tumor sizes ≥500 mm^3^ were considered the experimental endpoint. Animals with skin surface ulcerations were excluded from experiments and sacrificed immediately.

### Anti-CTLA4 antibody ELISA

Two-fold serial dilutions of the culture medium were incubated for 45 minutes in 96-well microtiter plates previously coated with 500 ng/well recombinant CTLA4 protein [16]. After washing, the wells were incubated with biotin-labeled anti-HA antibody (Roche, Mannheim Germany), followed by streptavidin-HRP (Jackson ImmunoResearch Laboratories) and finally 1-Step™ Ultra TMB-ELISA (Thermo Scientific, Rockford, IL) substrate for 30 min at room temperature. The absorbance of wells (405 nm) was measured with a microplate reader.

### Mouse cytokine array

Pieces of tumor were homogenized in Complete Lysis Buffer M (Roche) using TissueRuptor (Qiagen). The total protein concentration was determined using the Protein Assay reagent from Bio Rad. For each sample, 100 µg were assayed and the Cytokine Array Panel A (R&D Systems) was performed according to manufacturer's suggestions. For the development of the assay, ECL Plus (GE Healthcare) was used together with Amersham Hyperfilm ECL (GE Healthcare). The developed films were scanned and intensities were quantified with SigmaGel (Jandel Scientific, San Rafael, CA).

### Anti-CTLA4 mRNA

mRNA isolation from MMC-Rlx cells and qRT-PCR was performed as described recently [23]. cDNA was synthesized using the QuantiTect Reverse Transcription Kit (Qiagen). For PCR the SYBR Kit (Bioline, Taunton, MA) and the following primers were used

aCTLA4, fw 5′- ACC CCT CAC AAT CAC TGT CC -3′

aCTLA4, rev 5′- CAC CTG CAG GAA GAA CTG GT -3′

Anti-CTLA4 mRNA was equalized to levels of GAPDH mRNA measured in parallel in each sample. Ct values were calculated using the Sequence Detection System software (Applied Biosystems). The difference between the number of PCR cycles required for the two samples to reach a certain fluorescence signal shows how much of the mRNA of interest is present in the two samples relative to each other. Each cycle difference is equal to a fold difference of 2.

### qRT-PCR TGF-β1

Pieces of tumor were homogenized using TissueRuptor (Qiagen) and total RNA was purified with miRQURY RNA Isolation Kit (Exiqon, Woburn, MA). RNA concentration was measured on a NanoDrop ND-1000 (Thermo Scientific). Generation of cDNA was done with QuantiTect Reverse Transcription Kit (Qiagen) and the qPCR reaction was run, in triplicates, on a 7900HT Fast Real-Time PCR System (Applied Biosystems/Life Technologies) using the SensiMix SYBR Kit (Quantace, London, UK).

Primers (synthesized by Integrated DNA Technologies):

mTGFB1 fw 5′-GGCTACCATGCCAACTTCTG -3′

mTGFB1 rev 5′-CGCACAATCATGTTGGACA -3′

mTGFB2 fw 5′-TGAGGTGTGAATGCAAGGAG-3′

mTGFB2rev 5′-CAGTGAAGTGGAAGGGGAAA-3′

mTGFB3fw 5′-GCCATTTCCCTCCTACCCTA-3′

mTGFB3 rev 5′-CATCCATGATTCCCCAAAAA -3′

PCR was carried out as follows: after an initial 10-minute enzyme activation step at 95°C, 40 amplification cycles were run, each consisting of 95°C for 15 s and 60°C for 1 min. Lastly, a final elongation step was performed for two minutes at 60°C. Data was collected initially and after every incubation at 60°C. Anti-TGF-β mRNA was equalized to levels of GAPDH mRNA measured in parallel in each sample.

### Analysis of TILs

Three weeks after tumor cell transplantation, mice were sacrificed and tumors and spleen were harvested. Isolated MMC tumors were minced and filtered through a 70-µm cell strainer. Tumor infiltrating lymphocytes (TILs) were then isolated from tumor cells/erythrocytes by centrifugation in a Ficoll gradient. TILs as well as splenocytes from tumor-bearing mice were used for analysis of *Neu*-specific T-cells using a *Neu*-tetramer assay. The PE-labeled H-2Dq/R*NEU*420–429 (H-2D(q)PDSLRDLSVF) tetramer was obtained from the National Institute of Allergy and Infectious Diseases MHC Tetramer Core Facility (Atlanta, GA). Flow cytometry was performed with the following monoclonal antibodies (final concentration 5 µg/ml): anti-FoxP3-PE (clone FHK16s, eBiosciences, San Diego, CA), anti-CD4-PE, anti-CD8-PE, anti-CD8-FITC, anti-CD25-FITC (clone 7D4) (all BD Biosciences), anti-CD25-FITC (clone PC61.5; eBiosciences), and NK1.1-FITC (clone PK136, BD Biosciences), anti CD1d-PE (clone 1B1, BD Biosciences). All samples were treated with Fc-block (anti–CD16/CD32, BD Biosciences). Corresponding isotope controls yielded no significant staining.

### Immunohistochemistry for mouse tissue and organs

Tumors were embedded in Optimal Cutting Temperature (OCT) medium and frozen at −80°C. Sections were cut at a thickness of 8 µm and fixed in methanol∶aceton (1∶1 v/v) at −20°C for 10 min. Nonspecific binding was blocked by 2% non-fat dry milk in PBS for 20 min at RT. Primary antibodies were incubated at RT for 1 h. We used anti-CD1d-biotin (clone 1B1, BD Biosciences) and anti-NK1.1-FITC (clone PK136, BD Biosciences) antibodies. For histological assessment of autoimmune disease, mouse tissues and organs (heart, lung, brain, stomach, mesenterium, liver, kidney, muscle, skin) were fixed in 10% formalin and processed for hematoxilin and eosin staining. All samples were examined by two experienced pathologists for typical inflammation signs in a blind fashion. Immunohistochemistry for IgG on kidney sections was performed as described for tumor sections using a polyclonal, HRP-labeled, anti mouse IgG antibody (eBiosciences).

### Blood analysis

Mouse blood was analyzed using a HemaVet 950FS machine.

### Statistical analyses

Statistical significance of *in vivo* data was analyzed by Kaplan-Meier survival curves and logrank test (GraphPad Prism Version 4). Statistical significance of *in vitro* data was calculated by two-sided Student's *t*-test (Microsoft Excel). *P* values<0.05 were considered statistically significant. JMP statistical package was used to perform power analysis and determine the minimal number of animals per group. Using parameters of alpha = 0.05; power = 80%; effect size = 50% (80% chance of observing a difference of 50% in tumor size at a level of significance of 0.05), we arrived at a minimal group size of 5 for a comparison of two groups. Therefore, all experiments were performed at least once with 5 animals per group and, if required, repeated with additional animals until significance was achieved.

## Results

### Systemic application of anti-CTLA4 antibody

**Figure 1:**
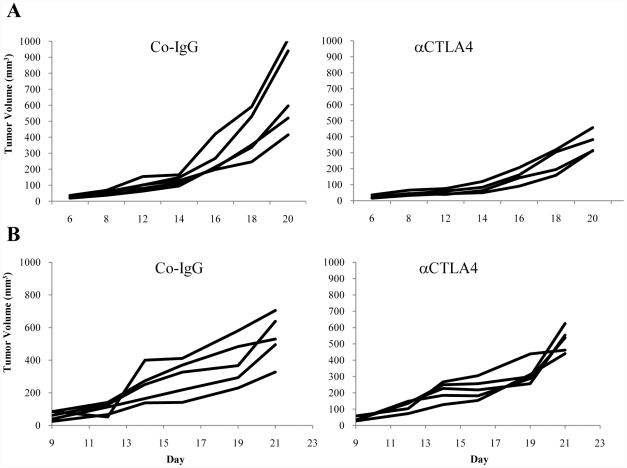
Effect of anti-CTLA4 antibody injection on MMC and TC-1 tumor growth. **A**) C57Bl/6 mice with subcutaneous TC-1 tumors were injected with 100 µg anti-CTLA4 and control IgG antibody intraperitoneally (i.p.) every other day. Treatment was started when tumor reached a volume of 50 mm^3^. Tumor volumes were measured thrice a week. Each line represents an individual animal. p<0.05 for time points after day 16. **B**) *neu*-transgenic mice with subcutaneous MMC tumors were treated as described in A). The difference between the two groups was not significant.

Clinically, antiCTLA4 antibodies are administered systemically. For our studies in mouse models, we used a monoclonal antibody against murine CTLA4 (4F10). Systemic administration of this antibody has been shown before to trigger tumor-destructive immune responses in several mouse tumor models [24], [25]. As in those studies, we started injecting anti-CTLA4 or control antibody when tumors reached a volume of 50 mm^3^. Injections were repeated every other day. While in the TC-1 tumor model, anti-CTLA4 injection significantly delayed tumor growth, it had no therapeutic effect in the MMC tumor model (Figs. 1A, B). These studies indicate that anti-CTLA acts differently in tolerized and non-tolerized tumor models.

### Expression of anti-CTLA4 antibody from tumor cells

While systemic anti-CTLA4 administration is technically straightforward, it is cost-extensive and also bears the risk of inducing auto-immune responses [16]. These problems can, in part, be addressed by gene therapy approaches resulting in intratumoral expression of genes encoding anti-CTLA4 antibodies. Expression of anti-CTLA4 antibodies inside the tumor has advantages over systemic administration. Presumably, at sites where the TAA levels are elevated, such as in the tumor microenvironment, peripheral tolerizing mechanisms must be enhanced relative to other tissues. To be most effective, the concentration of immune-stimulatory molecules should therefore be high in the tumor environment. Recently, an immunostimulatory effect of intratumoral expression of a gene encoding a secreted form of the anti-CTLA4 antibody has been shown in a model for autoimmune diabetes [26].

**Figure 2:**
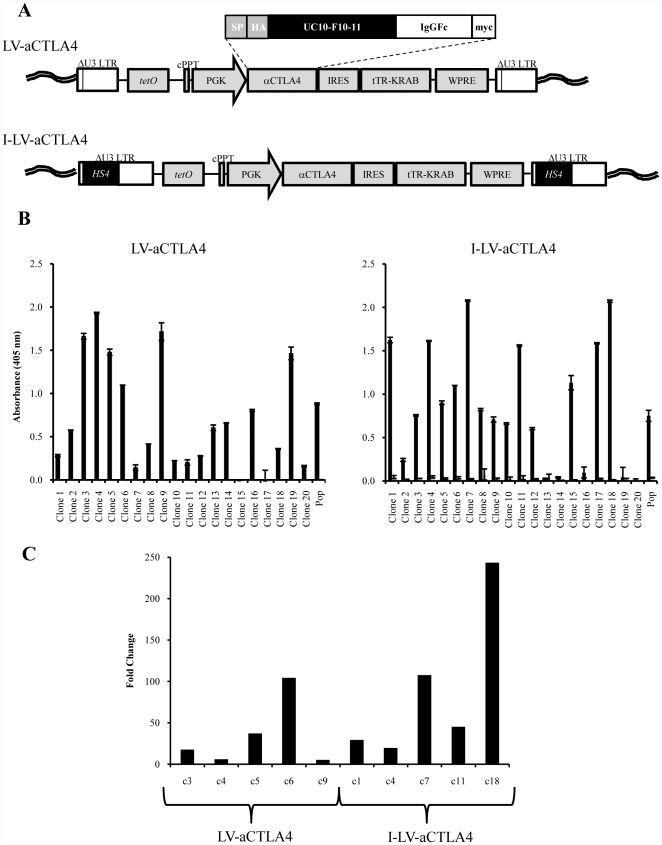
Lentivirus vectors expressing anti-CTLA4. **A**) Structure of integrated provirus genomes. The vectors contain the gene for the monoclonal antibody 4F10 (ATCC: UC10-4F10-11) specific to mouse CTLA4. The *anti-CTLA4* gene contains an immunoglobulin Vκ signal peptide, an HA epitope, the anti–CTLA4 scFv, the hinge, CH_2_ and CH_3_ domains of human IgG1, and a myc epitope. The *anti-CTLA4* gene is under the control of a tTR-KRAB system [18]. tRT-KRAB bound to tet-operator sequences represses promoters in the vicinity of 3-4 kb. Addition of Dox releases this repression. The vector also contains a central polypurine tract (cPPT) and a woodchuck hepatitis virus post-transcriptional regulatory element (WPRC). In the insulated vector version (I-LV-aCTLA4), a 0.4 kb cHS4 insulator element [43] is inserted into the 398 bp U3 promoter/enhancer deletion (U3Δ). Upon proviral integration into host genome, the U3 region containing the cHS4 is copied over to the 5′ LTR. **B**) Evaluation of anti-CTLA4 expression on protein level for clones derived after transduction of MMC cells with LV-aCTLA4 (upper panel) or I-LV-aCTLA4 (lower panel). Solid bars: Dox induced expression. Clones were treated with Dox and 24 h later anti-CTLA4 was measured by ELISA in culture supernatants. Empty bars (to the right side of solid bars): non-induced expression levels: supernatant from clones w/o Dox treatment were analyzed by ELISA. (Note, that these bars are not visible for LV-aCTLA4). Anti-CTLA4 levels in the corresponding populations of transduced cells (Pop) are shown on the right. **C**) Fold change of anti-CTLA4 mRNA levels after culture with or without doxycycline for 24 h measured. Clones that expressed the highest levels of anti-CTLA4 protein were included in this analysis. mRNA was isolated and subjected to qRT-PCR for GAPDH and anti-CTLA4 mRNA. Shown is the fold difference of GAPDH normalized anti-CTLA4 mRNA levels with and without Dox induction. Standard deviation was less than 10%.

To test our therapy approaches, we generated improved versions of lentivirus vectors. These vectors are self-inactivating (SIN), i.e. contain a deletion within the 3′LTR, which abolishes the LTR promoter activity (Fig. 2A). Because it had been shown that a chromatin insulator derived from the chicken globin locus control region DNase I hypersensitivity 4 region (cHS4) protects retrovirus vectors from chromosomal position effects of integration and from silencing, particularly in HSCs and their progeny [27], [28], we constructed an “insulated” SIN vector (I-LV-aCTLA4) by inserting the 0.4 kb cHS4 into the 3′ LTR. In the integrated I-LV-aCTLA4 provirus DNA, the transgene cassette is therefore flanked by two HS4 insulators (Fig. 2A, lower panel). A corresponding vector without cHS4 insulators was called LV-aCTLA4. The anti-CTLA4 antibody expression cassette can be activated by the addition of Dox, and Dox withdrawal ceases anti-CTLA4 antibody expression. This safety feature was built in to control potential side effects of anti-CTLA4 by expressing it only transiently. To functionally validate the vectors and assess the impact of the cHS4 insulators on position effects of integration, we infected MMC cells at an MOI of 1 cfu/cell and established clonal cultures by limited dilution. Anti-CTLA4 protein levels were measured in supernatants of the population (w/o subcloning) and 20 clones with and without Dox induction (Fig. 2B). There was no significant difference in induced anti-CTLA4 protein levels between I-LV-aCTLA4 transduced MMC cell clones and clones that were transduced with the non-insulated vector. As a more sensitive means to measure anti-CTLA4 expression we used qRT-PCR (Fig.2C). The increase in anti-CTLA4 mRNA levels upon Dox treatment was also not significantly greater in I-LV-aCTLA4 transduced clones compared to LV-aCTLA4 clones (p = 0.06). Overall, these data show that the inclusion of chromatin insulators into lentivirus vectors did not improve Dox mediated regulation of transgene expression.

**Figure 3:**
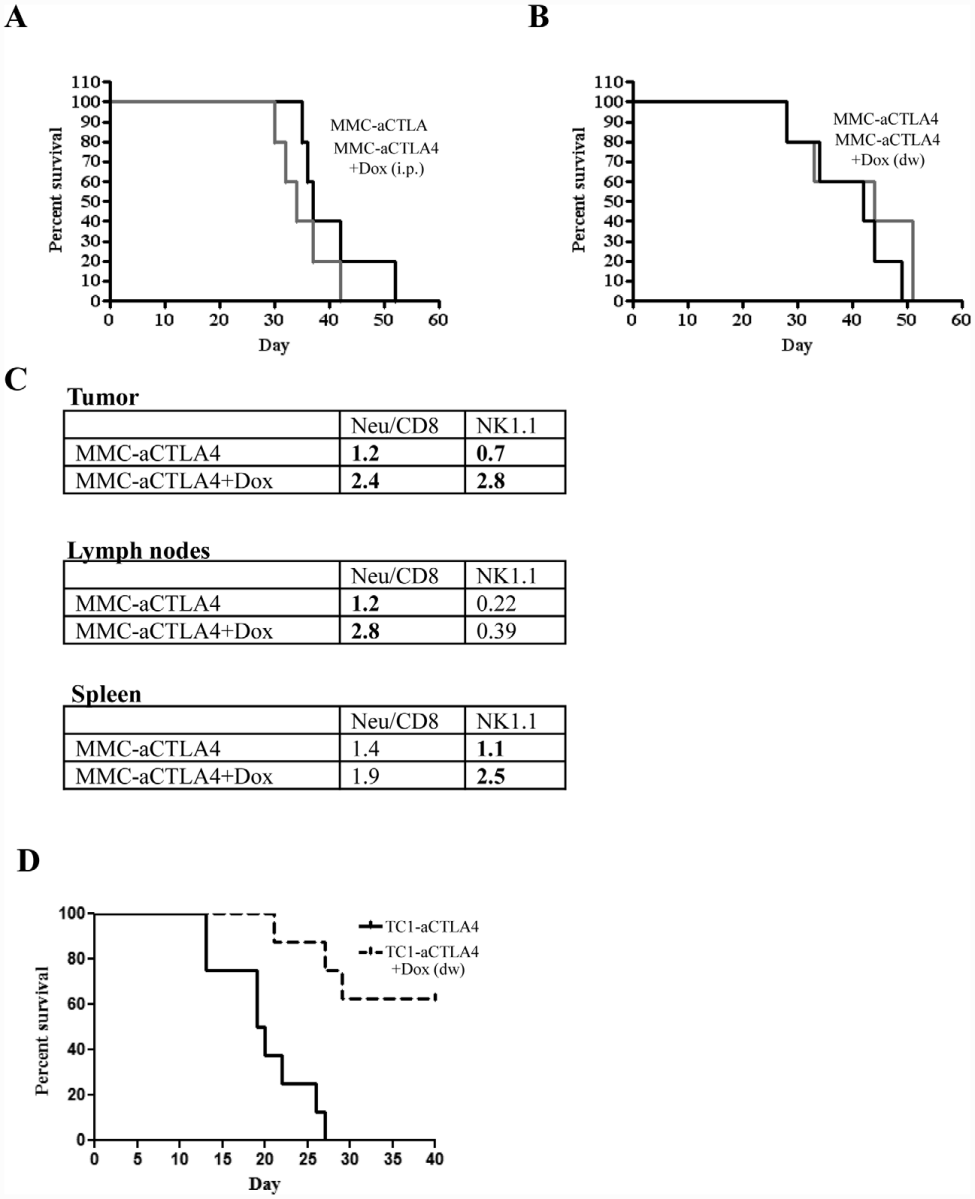
Growth and analysis of tumors derived from tumor cells transduced with LV-aCTLA4. A and B) MMC-aCTLA4 cells were injected and Dox treatment was started when tumors reached a volume of 50 mm^3^. Kaplan-Meier survival studies (cut-off is 1000 mm^3^). Dox was delivered with i.p. injection (A) or drinking water (B). N = 5. **C**) Flow cytometry of tumor infiltrating leukocytes and splenocytes in the MMC-aCTLA4 model. At day 30, tumors, tumor-draining lymph nodes and spleens were harvested. The percentages of *Neu* specific CD8+ T-cells were measured by tetramer assay. Shown are the average percentages of marked cells in all TILs, lymph node cells and splenocytes. Standard deviations were less than 10%. N = 3. **D**) TC-aCTLA4 cells were injected and Dox treatment was started when tumors reached a volume of 50 mm^3^. Kaplan-Meier survival studies (cut-off is 1000 mm^3^). Dox was delivered with drinking water. N = 5.

For therapy studies *in vivo*, we used an MMC cell clone that stably expressed anti-CTLA4 under Dox control (clone 4 generated from LV-aCTLA4 transduced MMC cells; see Fig. 2B). MMC-aCTLA4 cells were injected into *neu-*tg mice. When tumors reached a volume of 50 mm^3^, Dox was given either intraperitoneally (Fig. 3A) or in drinking water (Fig. 3B) to half of the mice. Induction of anti-CTLA4 expression *in vivo* was confirmed on the mRNA level by qRT-PCR and protein level by ELISA with tumor lysates (data not shown). Dox induction of anti-CTLA4 expression in MMC-aCTLA4 cells *in vivo* did not prolong survival in both models. Flow cytometry analysis revealed significantly higher percentage of *Neu*-specific CD8 cells in the tumor and tumor-infiltrating lymph nodes in the MMC-aCTLA4+Dox group compared to the corresponding group that did not receive Dox (Fig. 3C). Interestingly, Dox induced anti-CTLA4 expression appeared to increase the percentage of NK cells in the tumor and spleen. There was no significant difference in the number of CD4/CD25 cells, i.e Tregs.

For studies with the TC-1 model, we selected a TC1-aCTLA4 cell clone that produced similar anti-CTLA4 levels as the MMC-aCTLA4 clone upon Dox induction. In contrast to the study in the MMC model, anti-CTLA4 expression from TC-1 cells resulted in a significant delay in tumor growth (Fig. 3D). This is in agreement with an earlier study, in which we also showed that the anti-tumor effect is mediated by an increase of tumor-infiltrating IFNγ-producing CD8^+^ T cells [16]. Again these studies suggest that anti-CTLA4 has no therapeutic effect in tolerized tumor models, although it appears to increase the number of intratumoral effector T-cells.

### HSC based delivery of anti-CTLA4 gene

Because viral gene transfer to epithelial tumors is inefficient, we employed a new stem cell based approach to deliver the anti-CTLA4 gene to the tumor [20]. Both types of tumors have epithelial features including various intercellular junctions and extracellular matrix surrounding tumor nests. These physical barriers limit the efficacy of gene delivery using virus-based vectors [29], [30]. Because of this, stem cell based gene delivery approaches have been evaluated. In this context, the tropism of mesenchymal stem cells (MSC) for tumors has been exploited to deliver anti-tumor cytokine genes using *ex vivo* gene-modified MSCs [31]. Furthermore, based on the finding that monocytes/macrophages have the ability to migrate within tissues, even in hypoxic microenvironments, genetically modified monocytes/macrophages or progenitors have been used to delivery therapeutic genes to tumors [32]. We have previously developed an approach based on hematopoietic stem cells (HSCs) for *in vivo* gene delivery [20]. This approach is based on the fact that tumor cells secrete a number of chemokines that actively mobilize myeloid progenitors from the bone marrow and recruit them to the tumor stroma, where they differentiate into tumor-associated macrophages (TAMs). TAMs are critical for tumor survival as they produce factors that trigger/support tumor growth, neoangiogenesis, immune escape and stroma development. Our approach involves the *ex vivo* transduction of bone marrow derived HSCs with lentivirus vectors that express the transgene under control of a Doxycyline (Dox)-inducible transcription cassette, and the transplantation of these cells into myelo-conditioned recipients, where they engraft in the bone marrow and provide a long-term source of genetically modified cells that will home to tumors. This approach allows for efficient transgene delivery to the center of tumors. For example, in mice transplanted with HSCs transduced with a GFP expressing lentivirus vector, ∼5% of all cells in MMC tumors were GFP positive, whereby most of the transgene expressing cells were TAMs [20], [33]. In a recent study, we also showed that the inducible intratumoral expression of the peptide hormone relaxin after the transplantation of mouse HSCs transduced with a relaxin-expressing lentivirus vector, delayed tumor growth in the MMC-tumor model [20]. Here we used this “Trojan Horse” approach to deliver the anti-CTLA4 antibody gene to TC-1 and MMC tumors.

**Figure 4:**
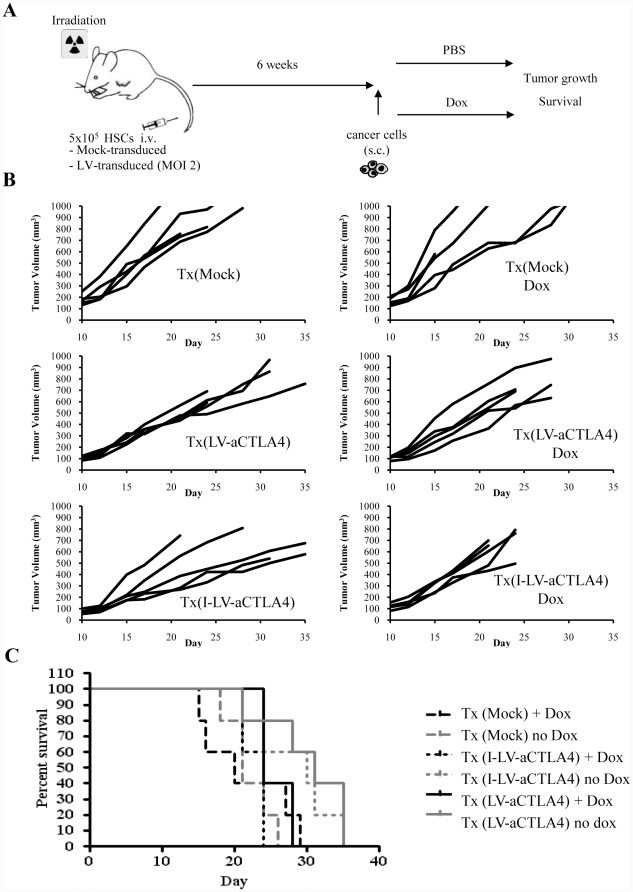
Therapy study with LV-aCTLA4/I-LV-aCTLA4 transduced HSCs and induction of transgene expression by Dox in drinking water. **A**) Scheme of the experiment: A total of 5×10^5^ LV-transduced mouse HSCs were transplanted into lethally irradiated *neu*-tg mice via tail injection. Six weeks after HSCs engraftment, MMC tumors were established via injection of 5×10^5^ MMC cells subcutaneously. Selected groups of mice received Dox in drinking water. **B**) Therapy study with mice that were transplanted with LV-aCTLA4 or I-LV-aCTLA4 transduced mouse HSCs; Tx(LV-aCTLA4, and Tx(I-LV-aCTLA4), respectively. Dox (0.2 mg/ml) was added to drinking water of selected groups starting at day 1 after MMC cell implantation. Each line represents an individual animal. **C**) Survival of MMC tumor bearing mice. The day tumors reached a volume of 900 mm^3^ represented the endpoint in Kaplan-Meier survival studies. N>5.

**Figure 5:**
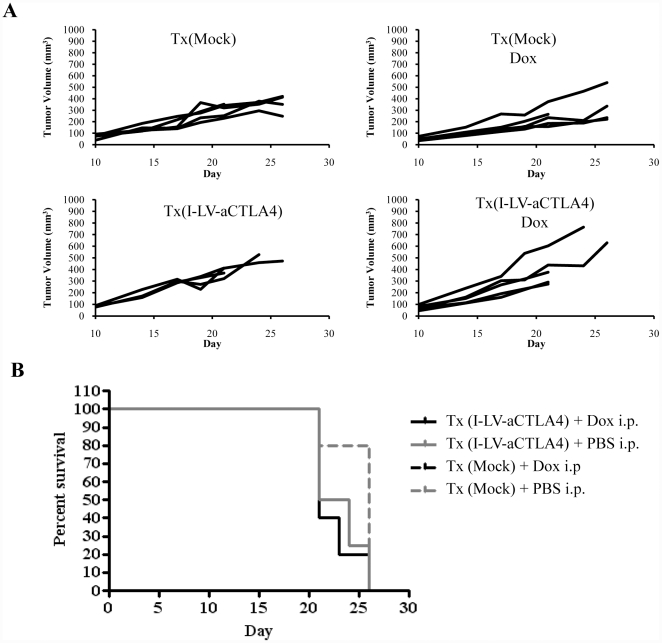
Therapy study with I-LV-aCTLA4 transduced HSCs and induction of transgene expression by Dox by intraperitoneal injection. Treatment scheme was as described in Fig.2. Mice received an i.p. injection of PBS or Dox (0.5 mg/mouse in 500 µl PBS) starting at day 7 after MMC cell transplantation and then every other day. **A**) Tumor volumes, **B**) Kaplan-Meier survival study (cut-off volume was 700 mm^3^). N>5.

As a source of HSCs, we used bone marrow cells from mice that were injected intravenously with 5-FU (150 mg/kg) two days prior to the collection of bone marrow. Bone marrow cells were cultured for three days and non-adherent cells (enriched for HSCs and primitive progenitors) were mock-transduced or transduced with LV-aCLTA4 or I-LV-aCTLA4 at an MOI of 1 cfu/cell. An aliquot of transduced HSCs was used to confirm successful transduction and anti-CTLA4 expression by qRT-PCR on cells cultured for 2 days. The rest of the cells were transplanted into lethally irradiated *neu*-tg mice. Six weeks later, subsequent to the bone marrow engraftment of genetically modified cells, mice were subcutaneously injected with MMC cells and Dox was given in drinking water to selected groups of animals (for a schematic of the experiment see (Fig. 4A). Mice were followed for 35 days. Animals were sacrificed before the end of the observation period if the tumors reached a volume of 1,000 mm^3^ or ulcerated. In explanted tumors, anti-CTLA4 mRNA was measured by qRT-PCR. In mice that received LV-aCTLA4 transduced HSCs, tumor anti-CTLA4 mRNA levels were 36-fold (+/−5) higher in Dox-treated mice compared to mice that received drinking water without Dox. There was no significant difference in anti-CTLA4 levels between LV-aCTLA4 and I-LV-aCTLA4 (Fig. 4B). Tumor volumes of individual mice and Kaplan-Meier survival studies are shown in Fig.4C. Unexpectedly, Dox treatment, i.e. induction of anti-CTLA4 expression in tumors, shortened the survival of mice (Tx(I-LV-aCTLA4) + Dox vs Tx(I-LV-aCTLA4): p = 0.029 and Tx(LV-aCTLA4) +Dox vs Tx(LV-aCTLA4): p = 0.095). Dox treatment of mock-transplanted mice did not affect MMC tumor growth [20]. To consolidate these findings, we performed a second experiment where Dox was given intraperitoneally to better control its delivery to mice (Fig. 5). As seen before, Dox-induced anti-CTLA expression did not exert therapeutic effects and shortened the life-span of animals.

**Figure 6:**
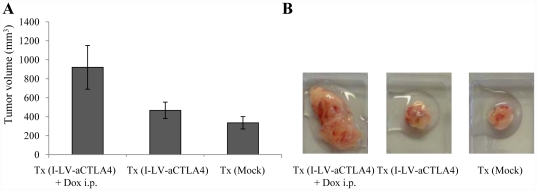
HSC-based anti-CTLA4 gene therapy in the TC-1 tumor model. Mice were treated as described in Fig. 4A. The experiment was terminated at day 18. **A**) Tumors were excised and measured. N = 5. **B**) Representative excised tumors.

In the TC-1 model, so far, injection of anti-CTLA4 or expression from TC-1 cells significantly delayed tumor growth. However, when we employed the HSC-based approach for *in vivo* expression of anti-CTLA4, we found a marked stimulation of TC-1 tumor growth upon Dox induction of anti-CTLA4 expression. Furthermore, tumor growth in the Tx(I-LV-aCTLA4 +Dox) group was more invasive involving subcutaneous muscle tissues. Because of this, it was impossible to measure tumor volumes over time. We therefore show the tumor volumes at the end of the observation period (day 35) (Fig. 6A). Representative explanted tumors are show in Fig.6B.

In summary, when the anti-CTLA4 gene was delivered using the HSC-based approach, it stimulated tumor growth in both tumor models.

### Mechanism of failure of HSC-based anti-CTLA4 therapy

**Figure 7:**
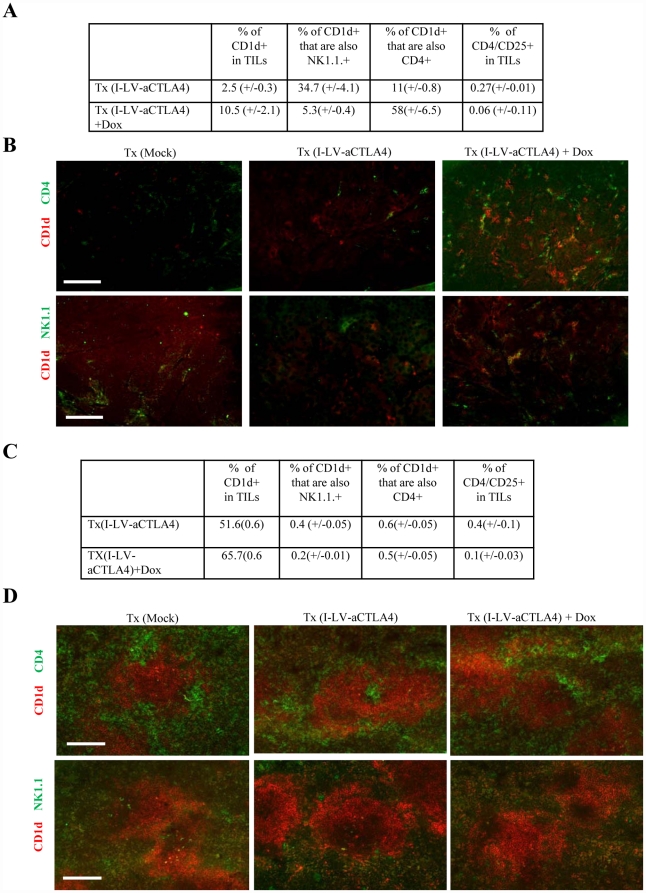
Analysis of immune cells in tumors and spleens of mice treated with I-LV-aCTLA4 transduced HSCs and induction of transgene expression by Dox by intraperitoneal injection. At day 28, tumors and spleens from Tx(I-LV-aCTLA4) and Tx(I-LV-aCTLA4)+Dox mice were harvested and analyzed. **A**) Tumors infiltrating leukocytes and splenocytes were subjected to flow cytometry for Cd1d, CD4, CD25, and NK1.1. N = 3. **B**) Tumors were sectioned and stained with antibodies against CD1d (red) and CD4 (green) (upper panel) or CD1d (red) and NK1.1 (green) (lower panel). Representative sections are shown. The scale bar is 40 µm. **C**) Flow cytometry analysis of splenocytes. N = 3. **D**) Immunofluorescence analysis of spleen sections. upper panel: CD1d (red) and CD4 (green); lower panel: CD1d (red) and NK1.1 (green).

To understand why anti-CTLA4 in these models did not suppress tumor growth, we performed flow cytometry and immunofluorescence analyses of immune cells in the spleen and the tumors. In addition to standard analyses for CD4 T-cells, NK cells, and Tregs, we also searched for changes in potential immunosuppressive cells. Among the latter is a specialized subset of NKT cells [10], [34], [35]. NKT cells are a unique T-cell subset expressing both TCR and NK cell receptors. Most NKT cells are restricted by the MHC class I–like molecule CD1d. In the mouse, most CD1d+ NKT cells are CD4^+^. An involvement of NKT in mediating tolerance to self-antigens and suppressing auto-immune inflammatory reactions has been reported in experimental and human autoimmune diseases [36]. A potential pathway that leads to immunosuppression involves the secretion of IL-13 by NKT cells and subsequent activation of Gr-1+CD11b+ myeloid suppressor cells, which in turn produce TGF-β1 [37]. In agreement with earlier studies [16], we found less CD4/CD25+ Tregs in I-LV-aCTLA+Dox tumors and spleen than in I-LV-aCTLA tumors (Figs. 7A and C). Importantly, however, both flow cytometry analysis of TILs (Fig.7A) and immunofluorescence analysis of tumor section (Fig.7B) showed significantly more CD1d+ cells in TILs of mice where anti-CTLA4 production was induced by Dox than in Tx(LV-aCTLA4) mice without Dox treatment and control mice that received mock transplantation (p = 0.026). The majority of CD1d+ cells in Tx(I-LV-aCTLA+Dox) tumors were CD4 cells (Fig.7A). Flow cytometry data were supported by costaining of tumor sections for CD1d and CD4 or NK.1.1 (Fig.7B). Notably, there was no significant difference in the composition and percentages of MMC tumor infiltrating leukocytes in Tx(I-LV-aCTLA) mice (without Dox induction) and mice that did not receive a bone marrow transplantation. Analysis of splenocytes of treated animals also showed a significant difference in the percentage of CD1d+ of I-LV-aCTLA+Dox and I-LV-aCTLA animals (Fig.7C, D). Figure 7 shows data from studies in the MMC tumor model. The outcome of studies in the TC-1 tumor model was similar.

**Figure 8:**
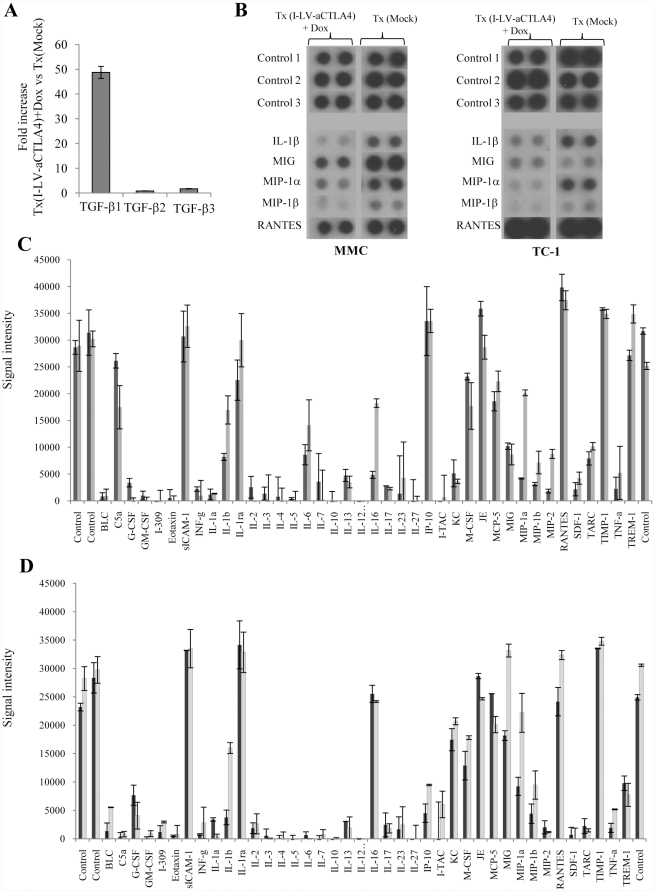
Analysis of intratumoral cytokine and chemokine expression. At day 28, TC-1 and MMC tumors from Tx(Mock) and Tx(I-LV-aCTLA4)+Dox were harvested. **A**) qRT-PCR for TGF-β RNA. Anti-TGF–β RNA was equalized to levels of GAPDH mRNA measured in parallel in each sample. Shown is the fold increase in Tx(I-LV-aCTLA4)+Dox vs Tx(Mock) groups in the TC-1 model. N = 3. The outcome in the MMC model was similar. **B–C**) cytokine proteome array. Tumor lysates were pooled and analyzed for the indicated cytokines using the Proteome Profiler from R&D Systems. **B**) Assembled images of dot blots for key cytokines from MMC and TC-1 tumors. **C**) (TC1) and **D**) (MMC). The plots were scanned and signal intensity was measured. BLC = CXCL13, I-309 = CCL1, eotaxin = CCL11, IP-10 = CXCL10, JE = CCL2, MIG = CXCL9, MIP1α = CCL3, MIP1β = CCL4, RANTES = CCL5, SDF-1 = CXCL12, TARC = CCL-17. The array includes positive controls provided by the manufacturer.

To further elucidate mechanisms of failure of HSC-based anti-CTLA4 therapy, we analyzed the expression of cytokines and chemokines in tumors. As TGF-β1 potentially mediates the immunosuppressive effect of CD11d+ NKT cells, we measured TGF-β1 mRNA by qRT-PCR in TC1 and TC1-aCTLA4+Dox tumors (Fig.8A). We found 49+/−5-fold higher concentrations of TGF-β1 mRNA in TC1-aCTLA4+Dox tumors than in TC-1 tumors. RNA levels of TGF-β2 and TGF-β3, i.e. cytokines that are not involved in immunosuppression did not differ between the two groups. Other cyto- and chemokines were analyzed by commercial proteome arrays. (These arrays did not cover TGF-β). The outcome of these studies was consistent for the TC-1 and MMC tumor models. In both models, HSC-mediated anti-CTLA4 expression resulted in a marked decrease of pro-inflammatory cyto- and chemokines in tumors, including IL-1β, MIG, MIP-1α, MIP-1β, and RANTES (Figs. 8B-D).

Notably, in all therapy studies, there were no signs of auto-immune responses such as changes in fur color or presence of inflammatory infiltrates on tissue sections of liver, lung or colon. Furthermore, immunohistochemistry staining for IgG complexes on kidney sections did not reveal abnormalities. Blood cell counts were normal in all groups of both models.

In summary, anti-CTLA4 expression from HSC progeny increases the percentage of CD4+/CD1d+ cells in tumors, which correlates with increased production of TGF-β1. Additionally, we found less cytokines that are involved in the activation of anti-tumor immune responses and attraction of T-cells in anti-CTLA4 expressing tumors.

## Discussion

Increased understanding of immune-regulatory mechanisms is required for the development of new immunotherapy agents that can modulate these signaling pathways and potentially break tumor tolerance. In this study, we report two findings: i) systemic delivery of anti-CTLA4 antibodies or intratumoral expression has different effects in non-tolerized and tolerized mouse tumor models and ii) HSC-mediated anti-CTLA4 expression triggers immunosuppressive mechanisms, which facilitate tumor progression.

There is an emerging picture that the same mechanisms that prevent autoimmunity also inhibit anti-tumor immune responses. The central problem in cancer immunotherapy is that most TAAs are non-mutated self-antigens that have triggered both central and peripheral tolerance. It is therefore important to test new immunotherapy approaches in mouse models, in which tolerance against the inoculated tumor and specific TAAs exist. Central tolerance is established by selection in the thymus: T-cells bearing T-cell receptors with high affinity for self-antigen are eliminated through apoptosis [38]. Additionally, peripheral T-cell tolerance is required to suppress the remaining auto-reactive T-cells in the periphery.

One goal of this study was to evaluate the anti-tumor effect of anti-CTLA4 in a mouse model that resembled key features of breast cancer in patients, most importantly tolerance to a TAA (*Neu*) and the presence of *Neu*-reactive T-cells. Anti-CTLA4 delivery as a protein or expression from gene-modified tumor cells were therapeutically efficacious in the non-tolerized TC-1 tumor model, but had no effect in the MMC-model, in spite of the fact that anti-CTLA4 expression from MMC tumors increased the number of *Neu*-specific T-cells. N*eu*-tg mice are tolerant to *Neu*. It has been discussed that in this model, subpopulations of high-avidity *Neu*-specific T cells are deleted centrally, whereas T-cells with lower avidity can leave the thymus but are subject to peripheral mechanisms of tolerance [22], [39]. This implies that tolerance to *Neu* involves both central and peripheral mechanisms. This finding suggests that the central tolerance mechanism must be overcome to enable intra-tumoral *Neu*-specific T-cells to kill tumor cells.

The model involving *ex vivo* transduced tumor cells is clinically not relevant. We therefore assessed an approach that would allow *in vivo* delivery of the anti-CTLA4 gene to the tumor. While viral gene delivery to epithelial tumors is inefficient after systemic application, recently a number of stem cell-based approaches have shown more promise. Our stem cell gene delivery approach is based on the *ex vivo* modification of HSCs, which home to the tumor after transplantation and deliver therapeutic transgenes to the tumor stroma. For HSC transduction, we used insulated SIN lentivirus vector with Dox-inducible transgene expression. In recent studies, we used the HSC-based approach to deliver the relaxin gene to tumors [20], [40]. In the MMC tumor model we showed that this approach facilitates pre-existing anti-tumor T-cells to control tumor growth. Furthermore, in xenograft models with Her2/*neu* positive breast cancer cells, HSC-mediated relaxin expression improved the anti-tumor efficacy of trastuzumab/Herceptin [33]. However, unexpectedly, when used for anti-CTLA4 gene delivery in this study, the HSC-gene approach was therapeutically detrimental in both the TC-1 and MMC- models. Anti-CTLA4 expression in these models triggered at least two reactions. It increased the percentage of CD1d+ NKT cells in tumors. These cells can theoretically activate myeloid suppressor cells, which in turn, produce TGF-β1. In support of this, we found ∼50-fold higher TGF-β1 mRNA levels in tumors of mice that received anti-CTLA4-HSC gene therapy. Additionally, HSC-based anti-CTLA4 gene delivery resulted in lower intratumoral levels of cytokines (e.g. IL-1β) and chemokines (e.g. MIG, MIP-1, RANTES) that stimulate immune responses. Proinflammatory cytokines such as IL-1β create a milieu in the tumor that is supportive for the activation of T- effector cells. Furthermore, a number of cells of the immune system, including pre-cDCs, show positive chemotaxis to CCL3 in a dose-dependent manner [41].

It remains in question, why expression of anti-CTLA4 from TC-1 cells had a positive therapeutic effect, while anti-CTLA4 expression after HSC transplantation into C57Bl/6 mice did not. Progeny of transplanted HSCs not only home to tumors and differentiate into TAMs, but also reconstitute spleen, thymus, and, potentially, macrophages in other tissues [20]. This implies that anti-CTLA4 is also expressed in non-tumoral tissue, which could have accounted for the unexpected outcome described above. Based on recent evidence that TAMs have a unique gene expression signature that distinguishes it from other tissue macrophages [42], we are currently working on TAM-specific expression systems to increase the tumor-specificity of transgene expression.

Overall, these findings suggest that stem cell based delivery methods, particularly for immuno-stimulatory genes, must ensure homing of stem cells to tumors or exclusive expression within tumors. The differences in outcomes between the tolerized and non-tolerized models also provide a potential explanation for the low efficacy of CTLA4 blockage approaches in cancer immunotherapy trials.
